# Polarization conversion when focusing cylindrically polarized vortex beams

**DOI:** 10.1038/s41598-016-0015-2

**Published:** 2016-12-05

**Authors:** Alexey P. Porfirev, Andrey V. Ustinov, Svetlana N. Khonina

**Affiliations:** 1 Samara National Research University, Technical Cybernetics Department, Samara, 443086 Russia; 2 Image Processing Systems Institute - Branch of the Federal Scientific Research Centre “Crystallography and Photonics” of Russian Academy of Sciences, Samara, 443001 Russia

## Abstract

Currently, cylindrical beams with radial or azimuthal polarization are being used successfully for the optical manipulation of micro- and nano-particles as well as in microscopy, lithography, nonlinear optics, materials processing, and telecommunication applications. The creation of these laser beams is carried out using segmented polarizing plates, subwavelength gratings, interference, or light modulators. Here, we demonstrate the conversion of cylindrically polarized laser beams from a radial to an azimuthal polarization, or vice versa, by introducing a higher-order vortex phase singularity. To simultaneously generate several vortex phase singularities of different orders, we utilized a multi-order diffractive optical element. Both the theoretical and the experimental results regarding the radiation transmitted through the diffractive optical element show that increasing the order of the phase singularity leads to more efficient conversation of the polarization from radial to azimuthal. This demonstrates a close connection between the polarization and phase states of electromagnetic beams, which has important implications in many optical experiments.

## Introduction

Phase singularities of a scalar field, which include vortex phases and phase jumps, are important features of various types of waves^1, 2^. Vector fields also exhibit a variety of polarization singularities^3, 4^. The spin angular momentum of photons was detected a long time ago^5–7^, and its interrelation with the orbital angular momentum has been discussed in several recent reviews^2, 4, 8–14^. Light beams with defined phase and polarization features are important for many applications, including optical manipulation^15–17^, microscopy^18–20^, materials processing^21–24^, and telecommunications^25–27^. Cylindrically polarized optical beams, which may have a radial or azimuthal polarization, have attracted the most attention from researchers because of their special properties^12^.

In some applications, such as STED (Stimulated Emission Depletion Microscopy) methods^20^, it is important to use a specific combination of laser beam polarization and spatial properties. In other applications, a desired state of polarization during the propagation of the beam must be retained, for example, to increase network throughput by using fibre modes that carry orbital angular momentum^27^. Polarization distribution control of the laser radiation enables some unique methods, like the selective excitation of an anisotropic molecule, focusing on a size smaller than the diffraction limit, and the fabrication of periodic nanostructures with femtosecond laser light^22, 23, 28^.

The conversion of polarization type can take place when beams with a phase singularity are tightly focused^29–31^. The transfer of angular momentum from the spin degree of freedom (which is related to the state of polarization) to the orbital (which is relevant to the phase distribution) degree of freedom can also occur in anisotropic media^32–35^. The interaction of the polarization singularities and phase singularities is used to detect the polarization state of the laser beam^36, 37^. Note that a complete differentiation of polarization types is only possible when the light is sharply focused^37^.

In this paper, we demonstrate the conversion of polarization type in a cylindrically polarized laser beam by introducing a higher-order vortex phase singularity. Earlier, a polarization distribution change for a vortex radially polarized beam in the propagation was shown^12, 38, 39^. However, the effects of a higher-order vortex phase in a cylindrical vector beam have not been previously studied. Here, the phenomenon of an orthogonal transformation in polarization is described empirically for the first time. The effect of the conversion of an azimuthally polarized beam to a radially polarized beam, and vice versa, is apparent even with weak focusing (in the paraxial case). A theoretical model for this phenomenon in focused cylindrical vector beams using the Debye approximation is also presented. In addition, a numerical study was performed, and the experimental results fully confirmed the theoretical predictions.

## Results

### Theoretical analysis of polarization conversion

**Figure 1:**
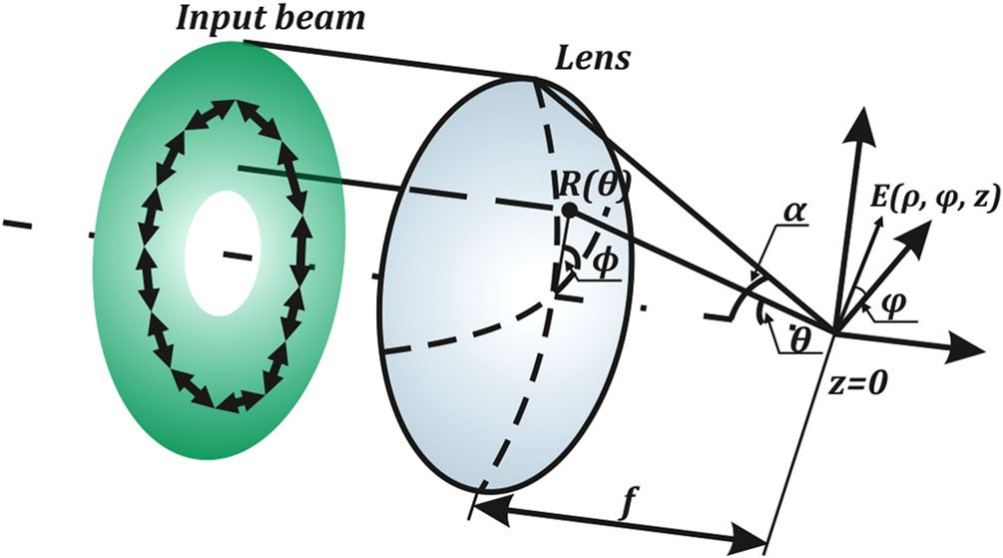
Vector Debye theory: focusing an azimuthally polarized beam through a lens of focal length *f* and maximum azimuthal angle *α*.

The sharp focusing of beams cannot be handled by the paraxial approximation and is usually solved by the Debye method^40^. The vector generalization of Debye theory is able to explain the behaviour of the polarization and intensity distributions of the electromagnetic field in the focal region. According to this method, the field distribution in the focal region is formed by wave rays that converge inside the cone bounded by the aperture of the optical system. Using the Debye approximation^41, 42^ for tight focusing, we obtain the following expression for the transverse components in the case of focusing an azimuthally polarized beam (as in this case, with no longitudinal component), having an *m*-th order vortex phase:

1 $$\begin{array}{rcl}{{\bf{E}}}_{m,\perp }^{az}(\rho ,\varphi ,z) & =&({E}_{m,\rho }^{az}(\rho ,\varphi ,z){E}_{m,\phi }^{az}(\rho ,\varphi ,z))\\&=&\frac{{i}^{m+1}kf{e}^{im\varphi }}{2} \times {\int }_{0}^{\alpha }R(\theta )T(\theta )\left({J}_{m+1}(k\rho \,\sin \,\theta )+{J}_{m-1}(k\rho \,\sin \,\theta )\right.\\ &&\left.-\,i\left[{J}_{m+1}(k\rho \,\sin \,\theta )-{J}_{m-1}(k\rho \,\sin \,\theta)\right]\right)\\  && \times \,\sin \,\theta \,\exp (ikz\,\cos \,\theta )d\theta ,\end{array}$$

where (*ρ*, *φ*, *z*) are the cylindrical coordinates in the focal region, (*θ*, *ϕ*) are the spherical angular coordinates of the focusing system’s output pupil, *α* is the maximum value of the azimuthal angle related to the system’s numerical aperture, *R*(*θ*) is the incident beam, *T*(*θ*) is the pupil’s apodization function (equal to $\sqrt{\cos \,\theta }$ for aplanatic systems), $k=\frac{2\pi }{\lambda }$ is the wavenumber, *λ* is the wavelength, and *f* is the focal length (see Fig. 1).

Equation (1) shows that the azimuthal polarization is retained only when focusing in the absence of a vortex phase (*m* = 0). When a vortex phase is present, part of the energy of the azimuthal component will be transferred to the orthogonal radial component. Let us determine the situation under which this transformation be most pronounced. To simplify the analysis, we consider the limits of integration in Eq. (1) to reflect a narrow ring *θ* ∈ [*α*_1_, *α*_2_], denoting the middle of the ring as ${\theta }_{c}\in \frac{({\alpha }_{1}+{\alpha }_{2})}{2}$.

Then, the ratio of the intensities of the orthogonal components in the focal region can be estimated by the following expression:

2 $${\eta }_{m}^{az\to rad}=\frac{{|{E}_{m,\rho }^{az}(\rho ,\varphi ,z=\mathrm{0})|}^{2}}{{|{E}_{m,\varphi }^{az}(\rho ,\varphi ,z=\mathrm{0})|}^{2}}\approx \frac{{|{J}_{m+1}(k\rho \sin {\theta }_{c})+{J}_{m-1}(k\rho \sin {\theta }_{c})|}^{2}}{{|{J}_{m+1}(k\rho \sin {\theta }_{c})-{J}_{m-1}(k\rho \sin {\theta }_{c})|}^{2}}\mathrm{.}$$

From Eq. (2), it is obvious that the intensity of the radial component will be greater than the azimuthal one when the following is true:

3 $${J}_{m+1}(k\rho \,\sin \,{\theta }_{c}){J}_{m-1}(k\rho \,\sin \,{\theta }_{c})\,> 0.$$

We can define the boundary of the region where the radial polarization dominates over the azimuthal with the following radius:

4 $${\rho }_{m}=\frac{{j}_{m-\mathrm{1,1}}}{k\,\sin \,{\theta }_{c}},$$

where *j*_*v*,1_ is the first zero of the *v*-th Bessel function of the first kind.

When |*m*| ≥ 2 at the central region of the focal plane, a radial, not azimuthal, polarization will be created. As the order of the vortex phase increases, this region will increase in size. Note also that the region of conversion shrinks as the numerical aperture of the focusing system increases (*θ*_*c*_ → 90°). That is, in the paraxial case with a weak focus, this effect will be more significant.

Let us now consider the following incident optical beam:

5 $$R(\theta )=\exp (-\,\frac{{\sin }^{2}\theta }{{\sin }^{2}\sigma })\frac{\sin \,\theta }{\sin \,\alpha },$$

where *σ* is the angular width of the waist of a Gaussian beam, and sin *α* is the numerical aperture of the focusing device.

Using certain approximations, including $T(\theta )\approx \frac{\cos \,\theta }{\sqrt{\cos \,\frac{\alpha }{2}}}$, we can transform the integral in Eq. (1) for a beam described by Eq. (5) into a form with tabular integrals^43^ and calculate it analytically.

Using the notation *x* = *kρ* sin *σ*, after mathematical transformations we obtain:

6 $${E}_{m,\rho }^{az}(x)\approx \frac{\sqrt{\pi}{\sin }^{3}\sigma}{4\,\sin \,\alpha \sqrt{\cos (\alpha/2)}}\times m\,\exp (-\frac{{x}^{2}}{8})[{I}_{(m-1)/2}(\frac{{x}^{2}}{8})-{I}_{(m+1)/2}(\frac{{x}^{2}}{8})],$$

7 $$\begin{array}{rcl}{E}_{m,\varphi }^{az}(x) & \approx &\frac{i\sqrt{\pi }{\sin }^{3}\sigma }{4\,\sin \,\alpha \sqrt{\cos (\alpha /2)}}\times \exp (-\,\frac{{x}^{2}}{8})\\  & & \times \left\{m[{I}_{(m-1)/2}(\frac{{x}^{2}}{8})+{I}_{(m+1)/2}(\frac{{x}^{2}}{8})]\right.\\  & & \left.-\frac{{x}^{2}}{2}[{I}_{(m-1)/2}(\frac{{x}^{2}}{8})-{I}_{(m+1)/2}(\frac{{x}^{2}}{8})]\right\}.\end{array}$$

In this analysis, we do not take into account factors that do not depend on *x* and *m* because the module for both components is the same. The radial component ${E}_{m,\rho }^{az}(x)$ is always positive for *m* > 0, and the azimuthal one ${E}_{m,\varphi }^{az}(x)$ changes sign once in this area. Because, for a constant argument, the function *I*_*m*_(·) decreases with increasing order, then we can show that in the region with positive values of ${E}_{m,\varphi }^{az}(x)$, the radial component is greater than the azimuthal component.

Moreover, the inequality ${E}_{m,\rho }^{az}(x)>  |{E}_{m,\varphi }^{az}(x)|$ holds over a wider range. As proof, in Eq. (7), there is a difference of functions ${I}_{\nu }(\cdot )$ with orders that differ by one. The recurrence relation for orders that differ by two is known: ${I}_{\nu -1}(y)-{I}_{\nu +1}(y)=2\nu {I}_{\nu }(y)/y$. We use the monotonic continuity of the modified Bessel functions to write the following as an approximation:

8 $${I}_{m\mathrm{/2}-1/2}(\cdot )-{I}_{m\mathrm{/2}+\mathrm{1/2}}(\cdot )\approx \frac{[{I}_{m\mathrm{/2}-1}(\cdot )-{I}_{m\mathrm{/2}+1}(\cdot )]}{2}\approx \frac{4m}{{x}^{2}}{I}_{m\mathrm{/2}}(\cdot )\mathrm{.}$$

By substituting Eq. (8) into Eq. (7), we obtain:

9 $${E}_{m,\rho }^{az}(x)\propto m[{I}_{(m-1)/2}(\frac{{x}^{2}}{8})-{I}_{(m+1)/2}(\frac{{x}^{2}}{8})],\\ {E}_{m,\phi }^{az}(x)\propto m[{I}_{(m-1)/2}(\frac{{x}^{2}}{8})-2{I}_{m\mathrm{/2}}(\frac{{x}^{2}}{8})+{I}_{(m+1)/2}(\frac{{x}^{2}}{8})]\mathrm{.}$$

The ratio of the intensities, similar to Eq. (2), will be the following:

10 $$\begin{array}{rcl}{\eta }_{m}^{az\to rad} & = & \frac{{|{E}_{m,\rho }^{az}(\rho ,\varphi ,z)|}^{2}}{{|{E}_{m,\varphi }^{az}(\rho ,\varphi ,z)|}^{2}}\approx \frac{{({I}_{(m-1)/2}-{I}_{(m+1)/2})}^{2}}{{({I}_{(m-1)/2}-2{I}_{m\mathrm{/2}}+{I}_{(m+1)/2})}^{2}}\\  & = & \frac{{[({I}_{(m-1)/2}-{I}_{m\mathrm{/2}})+({I}_{m\mathrm{/2}}-{I}_{(m+1)/2})]}^{2}}{{[({I}_{(m-1)/2}-{I}_{m/2})-({I}_{m/2}-{I}_{(m+1)/2})]}^{2}}.\end{array}$$

Because, as mentioned, the function *I*_*v*_(·) decreases with increasing order when the argument is held constant, we obtain ${\eta }_{m}^{az\to rad}> 1$.

When focusing a radially polarized field possessing an *m*-th order vortex phase, we obtain the following equation(in the paraxial case, we can consider only the transverse components):

11 $$\begin{array}{rcl}{{\bf{E}}}_{m,\perp }^{rad}(\rho ,\varphi ,z) & =&\left(\begin{array}{l}{E}_{m,\rho }^{rad}(\rho ,\varphi ,z)\\ {E}_{m,\phi }^{rad}(\rho ,\varphi ,z)\end{array}\right)=\frac{{i}^{m+1}kf{e}^{im\varphi }}{2}\\  & & \times {\int }_{0}^{\alpha }R(\theta )T(\theta )\left(\begin{array}{l} i\left[{J}_{m+1}(k\rho \,\sin \,\theta )-{J}_{m-1}(k\rho \,\sin \,\theta )\right]\\ {[{J}_{m+1}(k\rho \,\sin \,\theta )+{J}_{m-1}(k\rho \,\sin \,\theta )]}\end{array}\right)\\  & &\times \,\cos \,\theta \,\sin \,\theta \,\exp (ikz\,\cos \,\theta )d\theta.\end{array}$$

In the paraxial case, we can consider only the transverse components. In this way, we obtain a situation opposite to the one that was discussed above. That is, the radial polarization is transformed into an azimuthal polarization. This effect gets stronger as the order of the optical vortex increases:

12 $${\eta }_{m}^{rad\to az}=\frac{{|{E}_{m,\rho }^{rad}(\rho ,\varphi ,z=0)|}^{2}}{{|{E}_{m,\phi }^{rad}(\rho ,\varphi ,z=\mathrm{0})|}^{2}}\mathop{\to }\limits_{|m|\to \infty }\infty \mathrm{.}$$

### Numerical simulation

This section presents the results of simulating the polarization conversion using the Gaussian beam of Eq. (5) along with Eq. (1). For the numerical integration of Eq. (1), we chose the following parameters: sin *α* = 0.02, sin *σ* = 0.012.

**Figure 2:**
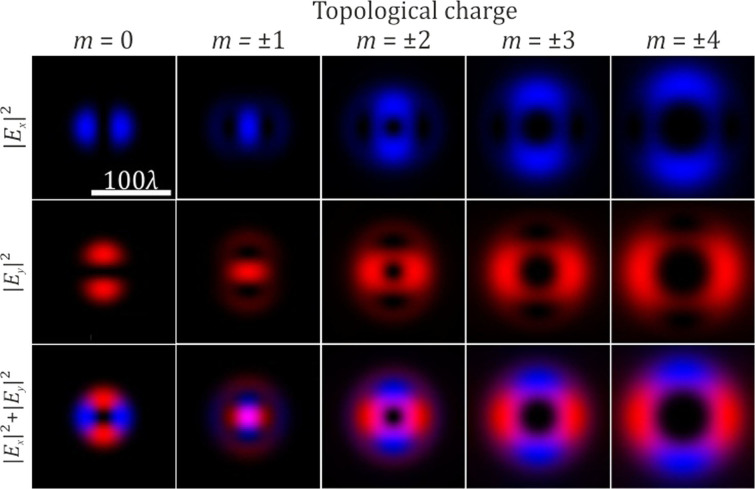
The distribution of the various components of the electric field in the focal plane for a radially polarized incident beam in the absence and presence of a vortex phase (the x-component is blue and the y-component is red).

Figure 2 shows the intensity distribution in the focal plane for the focused radially polarized beam described by Eq. (11) for $m=\overline{0,4}$. As observed in the absence of a vortex phase (*m* = 0), an initial radially polarized beam preserves the polarization in the focal plane. If we add a vortex phase of the first order, of either sign, in the focused radially polarized beam, a bright spot with circular polarization will be formed in the focal plane. If we add a vortex phase of a higher order, the conversion from radial polarization to azimuthal is observed.

**Figure 3:**
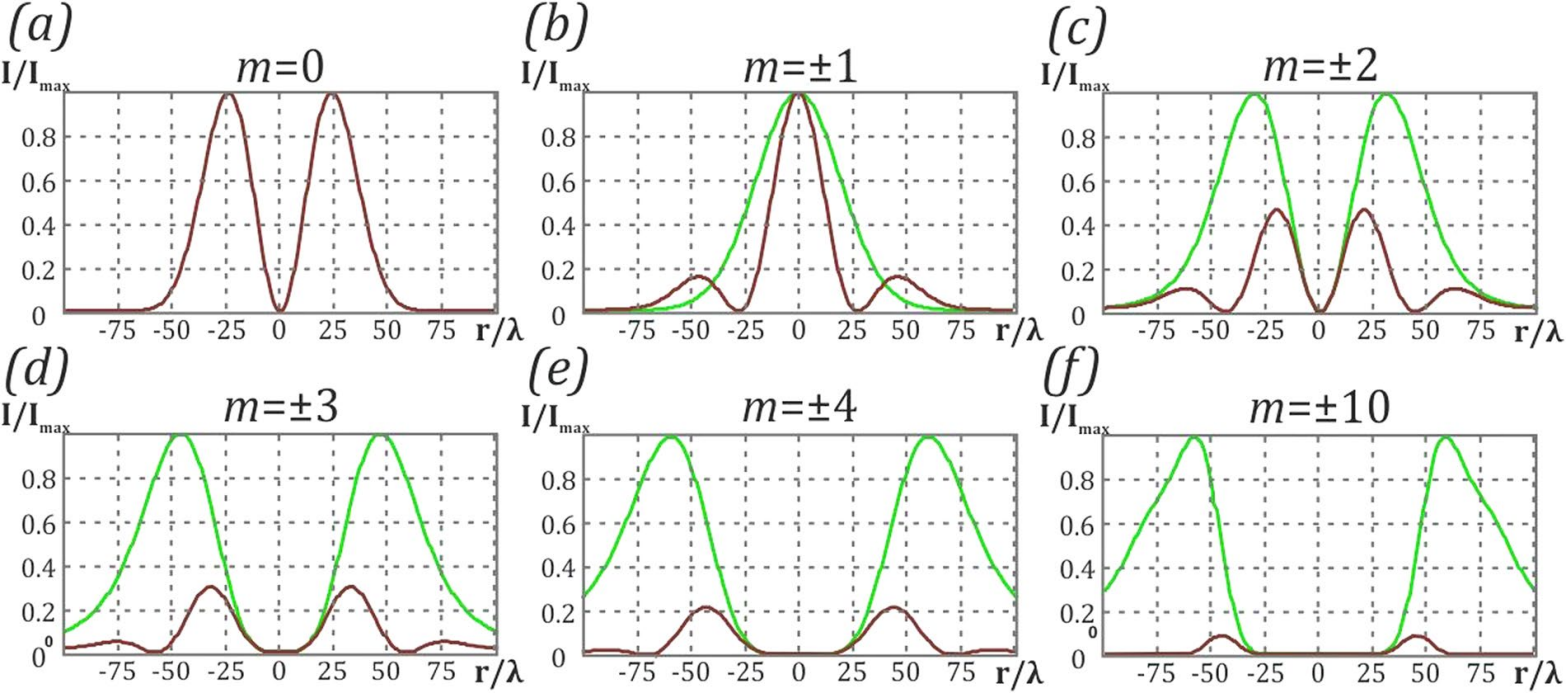
Distribution of the azimuthal and radial components of the electric field. (**a**) in the absence and (**b**–**f**) in the presence of a vortex phase: (**b**) *m* = ±1, (**c**) *m* = ±2, (**d**) *m* = ±3, (**e**) *m* = ±4, (**f**) *m* = ±10. The radial component is denoted by brown lines and the azimuthal component is denoted by green lines.

Figure 3 shows the focal distribution of the azimuthal and radial components of the electric field to quantitatively estimate the degree of conversion. We calculated the coefficient ${\eta }_{m}^{rad\to az}$ (see Eq. (12)) for different values of *m* to determine the degree of orthogonal polarization conversion. The values are ${\eta }_{m=0}^{rad\to az}=0$, ${\eta }_{m=\pm 1}^{rad\to az}=1$, ${\eta }_{m=\pm 2}^{rad\to az}=2.14$, ${\eta }_{m=\pm 3}^{rad\to az}=3.33$, ${\eta }_{m=\pm 4}^{rad\to az}=4.58$, ${\eta }_{m=\pm 10}^{rad\to az}=11.85$. As the order of the vortex increases, the contribution of the azimuthal component increases. However, even in the case of a 10-th order vortex, the radial component does not vanish completely.

## Experimental Results

**Figure 4:**
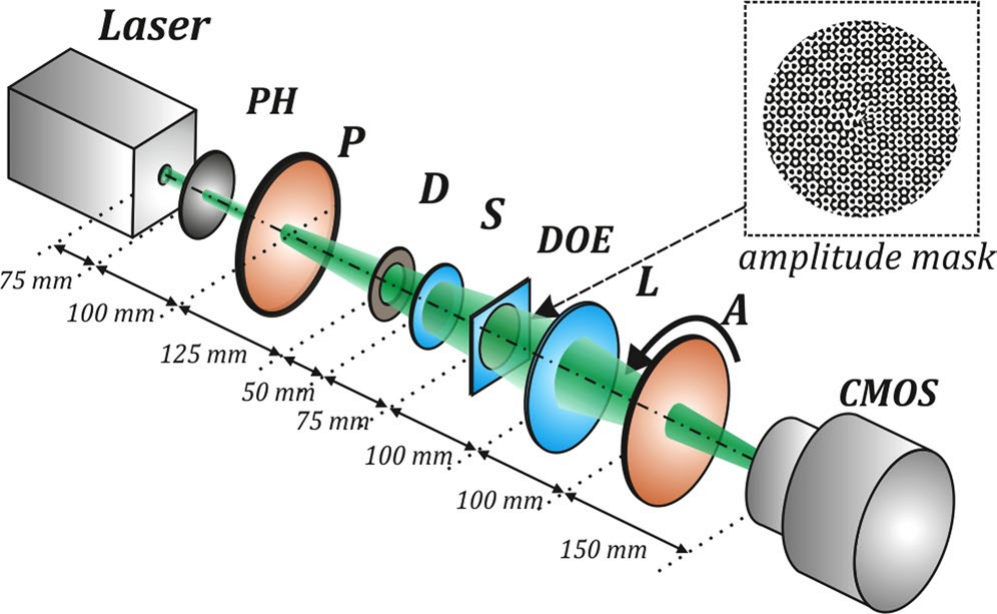
Experimental optical setup. The solid-state laser has an output wavelength *λ* = 532 nm, *PH* is a pinhole (100-*μ*m aperture), *P* is a polarizer, *D* is a diaphragm, *S* is an *S*-waveplate (radial polarization converter), *DOE* is an amplitude diffractive optical element forming a superposition of eight vortex beams with orders of ±1, ±2, ±3, and ±4, *L* is a lens with a focal length *f* = 150 mm, *A* is an analyser, and *CMOS* is a CMOS-video camera (LOMO TC-1000, 3664×2740 pixel resolution).

To investigate the polarization conversion experimentally, we utilized the experimental optical setup shown in Fig. 4. The output beam from a solid-state laser (*λ* = 532 nm) first passed through a pinhole *PH* (100-*μ*m aperture). Then, a polarizer *P* was used to obtain linearly polarized light with a predetermined polarization direction. A diaphragm *D* was used to separate the central spot of the Airy disk resulting from the wave diffraction of the pinhole. The *S*-plate^44^, oriented in the direction of polarization of the incident laser beam, converted the initially linearly polarized beam into a radially polarized beam. The resulting radially polarized laser beam illuminated the amplitude diffractive optical element (*DOE*), forming a superposition of eight vortex beams with orders ±1, ±2, ±3, and ±4 in different diffraction orders. The lens *L* (*f* = 150 mm) focused the laser beam on the camera sensor.

The inset in Fig. 4 shows the amplitude transmission function for the diffractive optical element. An amplitude mask is obtained by encoding^45^ the transmission function of the multi-order vortex DOE:

13 $${\rm{\Omega }}(x,y)=\sum _{n}\exp (i{m}_{n}\varphi )\exp [i(2\pi {u}_{n}x+2\pi {\nu }_{n}y)],$$

where *n* is an index of diffractive order, *m*_*n*_ is the topological charge of the vortex beam, and (*u*_*n*_, *v*_*n*_) are the carrier spatial frequencies.

**Figure 5:**
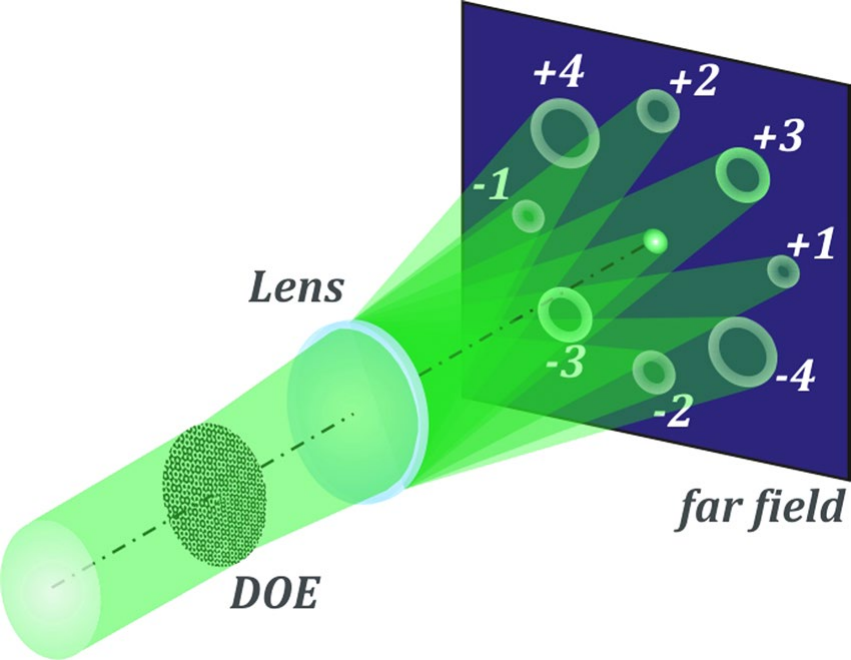
Operating principle of a multi-order vortex diffractive optical element.

The operating principle this DOE is shown in Fig. 5. The effect is the introduction of optical vortices of different orders in a radially polarized Gaussian beam, which initially does not have a vortex phase. Using a multi-order DOE, multiple optical vortices of different orders are formed, which allows the simultaneous measurement of the degree of polarization conversion at each spot.

**Figure 6:**
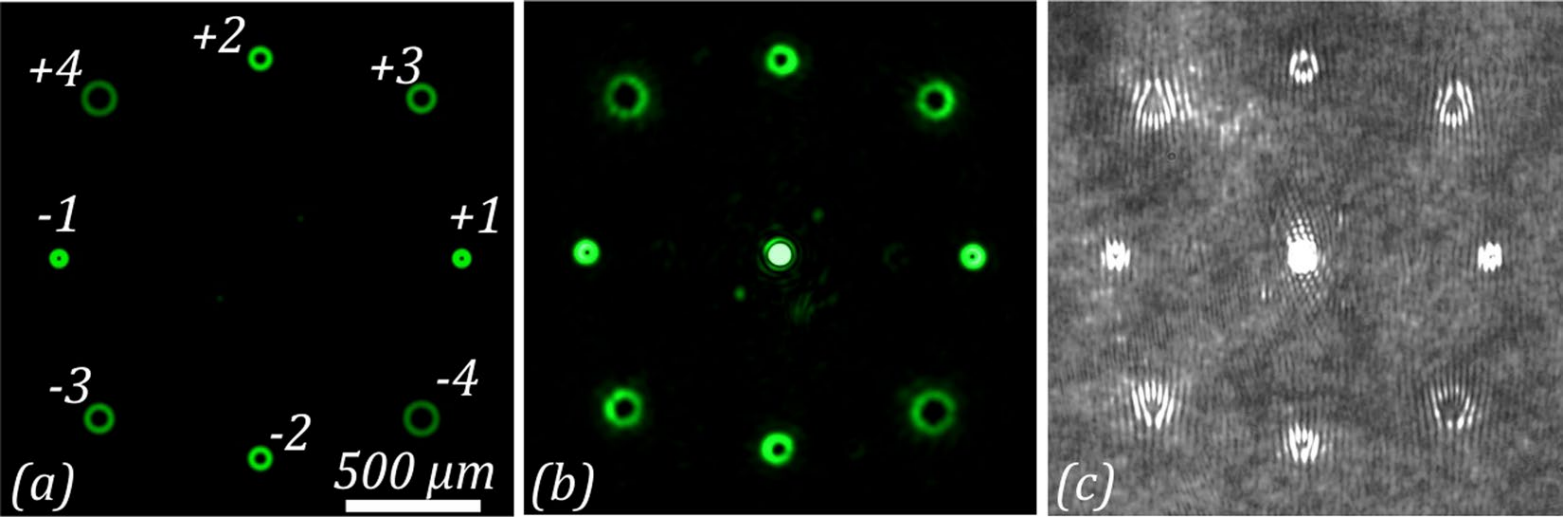
Diffraction pattern formed in the far-field region when passing the laser beam through the amplitude diffractive optical element, generating a superposition of eight vortex beams. (**a**) Simulation and (**b**) experimental result. (**c**) Experimentally obtained interference patterns of the generated vortex beams.

The diffraction pattern formed by the amplitude diffractive optical element is shown in Fig. 6. Figure 6a shows the simulated intensity distribution generated by the DOE. Because we utilized an amplitude DOE, the intensity of the zero diffraction order is too high in comparison with the intensity from the other diffraction orders. To clearly see the non-zero diffraction orders, we removed the zero diffraction order from the simulated picture. Figure 6b shows the intensity distributions obtained without the analyser. These experimentally obtained distributions show simultaneous interaction with eight optical vortices of different orders. Each of them is formed with a separate diffraction order at the corresponding location in the focal plane. In the paraxial case considered here, the displacement of the diffraction orders from the centre of the focal plane has no effect on polarization conversion. To confirm the order of the vortices, we recorded the interference pattern of the vortices with the Gaussian beam, resulting in characteristic fork fringes, as shown in Fig. 6c. The interference patterns confirm the order of the vortices to be *m* = ±1, ±2, ±3, and ±4, respectively.

**Figure 7:**
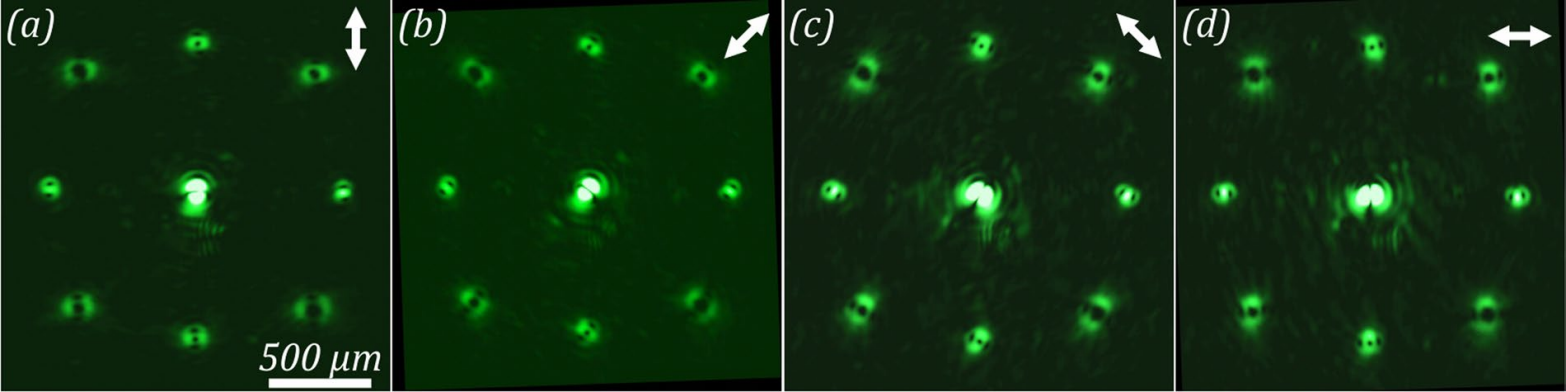
Diffraction pattern formed in the far-field region when passing the laser beam through the amplitude diffractive optical element, generating a superposition of eight vortex beams for different orientations of the analyser. (**a**) 0, (**b**) +45, (**c**) −45, and (**d**) 90 degrees.

**Figure 8:**
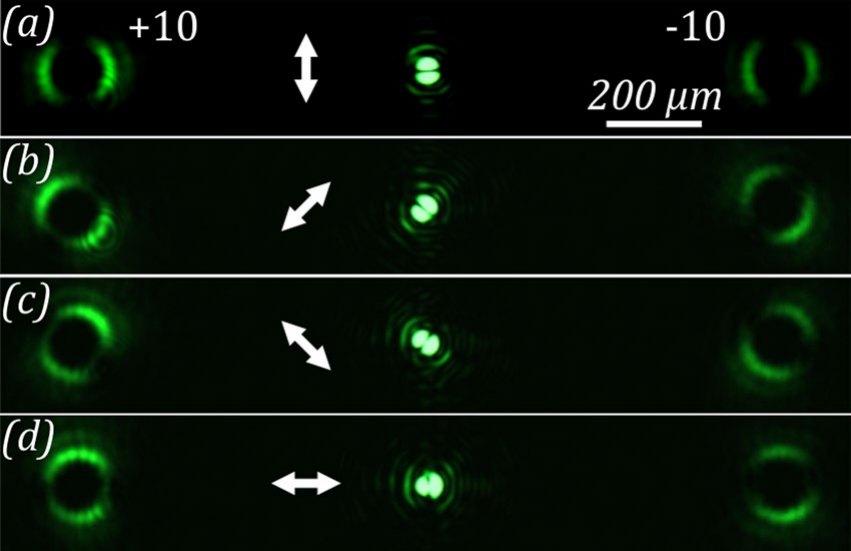
Diffraction pattern formed in the near-field region when passing the laser beam through the amplitude diffractive optical element, generating vortex beams with a topological charge *m* = ±10 for different orientations of the analyzer. (**a**) 0, (**b**) +45, (**c**) −45, and (**d**) 90 degrees.

Intensity distributions obtained with different orientations of the analyser are shown in Fig. 7. The analyser rotation angles are equal to ±45 and 90 degrees. The analyser orientation in these figures is represented by white arrows. In addition, the zero diffraction order, in which a laser beam has a topological charge of *m* = 0, gives an indication of the orientation of the analyser. As was predicted theoretically, in the case of optical vortices with *m* = ±2, ±3, and ±4 the conversion of radially polarized light into azimuthally polarized light is observed. For optical vortices with *m* = ±1, no conversion is observed. Figure 8 shows the intensity distributions formed in the far-field region using a diffractive optical element forming two vortex beams with topological charge *m* = ±10. It can be clearly observed that in this case, we obtain a laser beam with a nearly perfect azimuthal polarization, while the radial component decreases significantly. Thus, the experimental results are in good agreement with the simulation results presented above.

## Discussion

We conducted a theoretical analysis of the effect of sharply focusing a cylindrically polarized beam in the presence of an optical element with a vortex phase. Analytical expressions for the field in the focal region for radially and azimuthally polarized beams were obtained. In this way, we demonstrated the conversion of polarization state in cylindrically polarized laser beams by introducing a higher-order vortex phase singularity. In addition, a numerical study was performed. The experimental results are in good agreement with the simulation.

Our theoretical and experimental results show that increasing the order of the phase singularity leads to increased conversation of the radially polarized laser beam into an azimuthally polarized one. Our results demonstrate the close connection between of the polarization and phase states of electromagnetic beams. Specific combinations of the polarization and spatial properties of the laser beam are important for certain applications, such as telecommunication and materials processing. Bozinovic *et al*.^27^ presented multiplexing techniques that use the wavelength, amplitude, phase, and polarization of light to encode information. Taking into account the results presented here, it is necessary to use combinations of orthogonal polarization states and orbital angular momentum carefully. On the other hand, the effect of a phase vortex on the cylindrical polarization shown in our work will allow for a better understanding of the processes occurring during the interaction of laser radiation with matter, as in ablation^23^.

## Methods

### Model of focusing cylindrically polarized beams

In the Debye approximation of tight focusing, the cylindrical components of the electric field of a monochromatic electromagnetic wave are described by the following expression^31^:

14 $$\begin{array}{rcl}{\bf{E}}(\rho ,\phi ,z) & = & (\begin{array}{l}{E}_{\rho }(\rho ,\varphi ,z)\\ {E}_{\varphi }(\rho ,\varphi ,z)\\ {E}_{z}(\rho ,\varphi ,z)\end{array})=-\frac{if}{\lambda }{\int }_{0}^{\alpha }{\int }_{0}^{2\pi }B(\theta ,\phi )T(\theta )(\begin{array}{c}\cos \,\phi \,\cos \,\theta \\  -\,\sin \,\phi  \sin \,\phi \,\cos \,\theta   \cos \,\phi \\ -\sin \,\theta   0\end{array})({c}_{r}(\phi ) {c}_{\varphi }(\phi ))\\  &  & \times \exp [ik(\rho \,\sin \theta \,\cos (\phi -\varphi )+z\,\cos \,\theta )]\,\sin \,\theta d\theta d\phi ,\end{array}$$

where (*ρ*, *φ*, *z*) are the cylindrical coordinates in the focal region, (*θ*, *ϕ*) are the spherical angular coordinates of the focusing system’s output pupil, *α* is the maximum value of the azimuthal angle related to the system’s numerical aperture, *B*(*θ*, *ϕ*) is the transmission function, *T*(*θ*) is the pupil’s apodization function (equal to $\sqrt{\cos \,\theta }$ for aplanatic systems), $k=\frac{2\pi }{\lambda }$ is the wavenumber, *λ* is the wavelength, *f* is the focal length, *c*_*r*_(*ϕ*), *c*_*ϕ*_(*ϕ*) are the polarization coefficients of the incident radiation.

For vortex beams $B(\theta ,\phi )=R(\theta )\exp (im\phi )$ , Eq. (14) can be simplified as follows:

15 $${{\bf{E}}}_{m}(\rho ,\varphi ,z)=ikf{\int }_{0}^{\alpha }R(\theta )T(\theta ){{\bf{Q}}}_{m}(\rho ,\varphi ,\theta )\sin \,\theta \,\exp (ikz\,\cos \,\theta )d\theta ,$$

where the components of vector **Q**_**m**_(*ρ*, *φ*, θ) have a definite form for each specific polarization and are superpositions of the Bessel functions of different orders^31, 37^.

When interacting with a vortex phase, the azimuthal and radial polarization are of particular interest^12^. The expressions of **Q**_**m**_(*ρ*, *φ*, θ) for the corresponding polarization types are given below:

16 $${{\bf{Q}}}_{m}^{az}(\rho ,\varphi ,\theta )=-\,\frac{{i}^{m}{e}^{im\varphi }}{2}\left(\begin{array}{l}{J}_{m+1}(k\rho \,\sin \,\theta )+{J}_{m-1}(k\rho \,\sin \,\theta)\\-i\left[{J}_{m+1}(k\rho \,\sin \,\theta )-{J}_{m-1}(k\rho \,\sin \,\theta)\right.\\ 0\end{array}\right),$$

17 $${{\bf{Q}}}_{m}^{rad}(\rho ,\varphi ,\theta )=\frac{{i}^{m}{e}^{im\varphi }}{2}\left(\begin{array}{l}i\left[{J}_{m+1}(k\rho \,\sin \,\theta )-{J}_{m-1}(k\rho \,\sin \,\theta )\right]\,\cos \,\theta\\ i\left[{J}_{m+1}(k\rho \,\sin \,\theta ) -{J}_{m-1}(k\rho \,\sin \,\theta )\right]\,\cos \,\theta \\-2{J}_{m}(k\rho \,\sin \,\theta )\sin \,\theta\end{array}\right).$$

As observed from Eq. (16) and Eq. (17), the azimuthal polarization is the easiest to calculate because, in this case, only the transverse components of the field are nonzero.
